# Correlation Analysis of the Microbiome and Immune Function in the Lung-Gut Axis of Critically Ill Patients in the ICU

**DOI:** 10.3389/fmed.2022.808302

**Published:** 2022-03-18

**Authors:** Bin Liu, Ying Yu, Min Zhao, Kun Xiao, Peng Yan, Zhimei Duan, Kaifei Wang, Na Zhao, Jiabao Cao, Jun Wang, Lixin Xie

**Affiliations:** ^1^College of Pulmonary and Critical Care Medicine, Chinese PLA General Hospital, Beijing, China; ^2^Medical School of Chinese PLA, Beijing, China; ^3^CAS Key Laboratory of Pathogenic Microbiology and Immunology, Institute of Microbiology, Chinese Academy of Sciences, Beijing, China; ^4^University of Chinese Academy of Sciences, Beijing, China; ^5^Pharmaceutical Diagnostics, GE Healthcare, Beijing, China; ^6^Collaborative Innovation Center of Recovery and Reconstruction of Degraded Ecosystem in Wanjiang Basin Co-founded by Anhui Province and Ministry of Education, School of Ecology and Environment, Anhui Normal University, Wuhu, China

**Keywords:** microbiome, lung-gut axis, ICU, immune function, critically ill

## Abstract

**Objective:**

Critical illnesses in the intensive care unit (ICU) have been a global burden. We aimed to determine the correlation between the lung and gut in critically ill patients to find novel evidence of the lung-gut axis, which may be a new treatment for patients with critical illness in the ICU.

**Methods:**

We collected bronchoalveolar lavage specimens and fecal samples of 31 patients with critical illness within 24 h after admission. Metagenomics was used to detect lung and intestinal samples. Immune cells were detected by flow cytometry.

**Results:**

There are 86 common species in both lung and gut. The abundance of *Enterococcus faecium* is high in both the lung and gut of patients with critical illness in the respiratory intensive care unit (RICU). *Corynebacterium striatum* in the lung and gut is correlated with different immune cells. In addition, *C. striatum* in the lung and gut might share the same source, supporting the concept of a gut-lung axis in humans.

**Conclusions:**

The microbiome in the lung and gut showed a correlation to some extent, and *C. striatum* in the lung and gut might share the same source. In addition, the microbiome showed a correlation with immunity, indicating a potential therapeutic target in patients with critical illness. The lung-gut axis might play an important role in patients with critical illness in the RICU.

## Introduction

Critical illness in the intensive care unit (ICU) may cause tremendous global mortality and an enormous economic burden (1, 2). Despite improvements in antibiotic therapy, resuscitation strategies, and supportive care, critical illnesses, such as sepsis and acute respiratory distress syndrome (ARDS), are still leading to high mortality and increasing the risk of longer hospital stays (3). It is important to seek novel strategies for the diagnosis and treatment of critical illness in the ICU.

The human body hosts a massive and diverse microbiome. In the ICU, the microbiome of patients with critical illness may be affected by various factors, such as antibiotic use, mechanical ventilation, and diet changes, that could bring the microbiome out of balance (4, 5). The intimate relationship in the microbiome and immunological link between the gut and lung—the gut-lung axis—have drawn increasing attention (6, 7). The lung microbiome, which is altered in critical illnesses, such as sepsis, turns out to be selectively enriched with gut-associated bacteria (8).

In the present study, we used metagenomics to analyze the lung and gut microbiomes of patients with critical illness in the ICU to determine the correlation between the lung and gut microbiome-lung gut axes. The results showed that there were many common bacteria in the lung and gut microbiomes. *Corynebacterium striatum* in the lung and gut was correlated with different immune cells. In addition, a correlation analysis revealed that the lung and gut microbiomes were related to immunity in patients with critical illness.

## Methods

### Study Design and Patients

The present study was conducted in the Respiratory Intensive Care Unit (RICU) of College of Pulmonary and Critical Care Medicine, Chinese PLA General Hospital and was approved by the Ethic Committee of Chinese PLA General Hospital (ethics number: S2019-266-02); informed consent was obtained from the patient representative prior to the collection of lung samples *via* bronchoalveolar lavage, gut samples *via* feces, and blood samples from the vein. We included 31 patients older than 18 years admitted to the RICU with a length of stay longer than 24 h from September to December 2019.

### Specimen Collection and Processing

Bronchoalveolar lavage fluid (BALF) specimens were collected using a standardized bronchoscopy protocol. In short, patients received conscious sedation and nebulized lidocaine. The bronchoscope catheter was introduced through the nose or mouth and through the vocal cords and to the lobe of the lung. BALF was performed with instillation of between 120 and 200 ml of sterile isotonic saline, and 50–60 ml BALF was aspirated. Specimens were stored at −80°C until processing.

Fecal samples were collected within 24 h after admission and were stored at −80°C until processing.

Blood samples were collected within 24 h after admission and stored at 4°C until processing as soon as possible. Immune cells counts were measured by using the flow cytometry.

In our study, we collected the first BALF, feces, and blood of each patient in the first 24 h after admission.

### Data Analysis

A modified protocol for the Nextera XT DNA Library Preparation kit from Illumina was used for metagenome library construction. A KAPA mRNA Library Preparation kit was used for metagenome library construction. Sequencing was performed using the NovaSeq (PE150).

Bacterial raw reads were deduplicated using Kneaddata (version 0.7.3) to trim and filter low-quality sequences, as well as contaminate human reads with the human genome reference. Profiling the composition of microbial communities was performed using HUMAnN2 (version 2.8.1) by mapping reads to clade-specific markers (9), such as (1) screening with MetaPhlAn2 for taxonomic identification (10); (2) mapping reads to the annotated pangenomes of the identified species in the ChocoPhlAn database using Bowtie2 (11); and (3) translated search of unmapped reads against the UniRef90 protein reference database (12) using DIAMOND (13). StrainPhlAn was used to characterize the microbial compositions of the samples at the strain level.

### Statistical Analysis

The visualization of bioinformatic analyses based on the R package was performed under R version 4.0.1. First, the *vegan (version 2.5-7)* package was used to calculate the alpha diversity of the bacterial flora of the feces and lung, such as the Shannon and Simpson indices, which comprehensively reflect the richness and uniformity of the microorganisms. To show the microflora abundance between the two groups of feces and lungs at the phylum and species levels, we used the *graphics (version 4.0.1)* package to draw percentage stacked histograms to display the top 12 bacteria and applied different colors to represent them. A Venn diagram was applied to display the overlap of bacteria between the feces and lungs by the *Venn Diagram* (version 1.6.20) package. After normalizing the abundance of common bacteria in the feces and lung by *z*-score, the *pheatmap (version 1.0.12)* package was used to draw clustered heatmaps, selected “complete” for the clustering method, and used the “correlation” method for the column clustering distance. Principal component analysis (PCA) was performed on microflora traits to reduce dimensionality using dudi.pca in the *ade4 (version 1.7-16)* package. The first two latent variables were extracted to explain the difference, namely, first and second principal component axes (PC1 and PC2), the data were visualized through the *ggplot2 (version 3.3.2)* package, according to the PC1 and PC2 of each sample, and the *clusterSim (version 0.49-1)* package was used to determine the best cluster to join the clustering circle.

The Pearson correlation coefficient method was used to select the correlation coefficient > 0.75 between the microflora and the immune cells by the *psych (version 2.0.9)* package, and a *p* < 0.05 was considered a significantly related combination. All interactive relationship network visualizations were based on *Cytoscape (version 3.7.2)* (14). Labeled Heatmap in *WGCNA (version 1.69)* package plots heatmap with color legend, row and column annotation, and optional text within the heatmap.

## Results

### Clinical Characteristics of Patients

There were 31 patients with 31 paired sample of BALF-feces-blood. The average age of these patients was 63 years old. Mean SOFA score is 4.4 and mean APACHE II score is 23.8. The clinical characteristics of patients are shown in the Table 1.

**Table 1:** Clinical characteristics of 31 patients.

Male	21
Female	10
Mean age (years)	63
Mean albumin (g/L)	32
Mean creatinine (umol/L)	134
Mean total bilirubin (umol/L)	11.4
Mean d-dimer (ug/ml)	3.2
Mean count of platelet (10^9^/L)	205
Mean oxygenation index	235
Mean IgG (mg/dl)	1188
Mean CD4^+^ T cell/CD8^+^ T cell ratio	1.83
Mean SOFA score	4.4
Mean APACHE II score	23.8
patients with ARDS(n)	22
patients with Sepsis(n)	29

*IgG, immunoglobulin G; SOFA, sepsis-related organ failure assessment; APACHE II, Acute Physiology and Chronic Health Evaluation II; ARDS, acute respiratory distress syndrome*.

### The Composition of the Lung and Fecal Microbiome in Patients With Critical Illness

**Figure 1:**
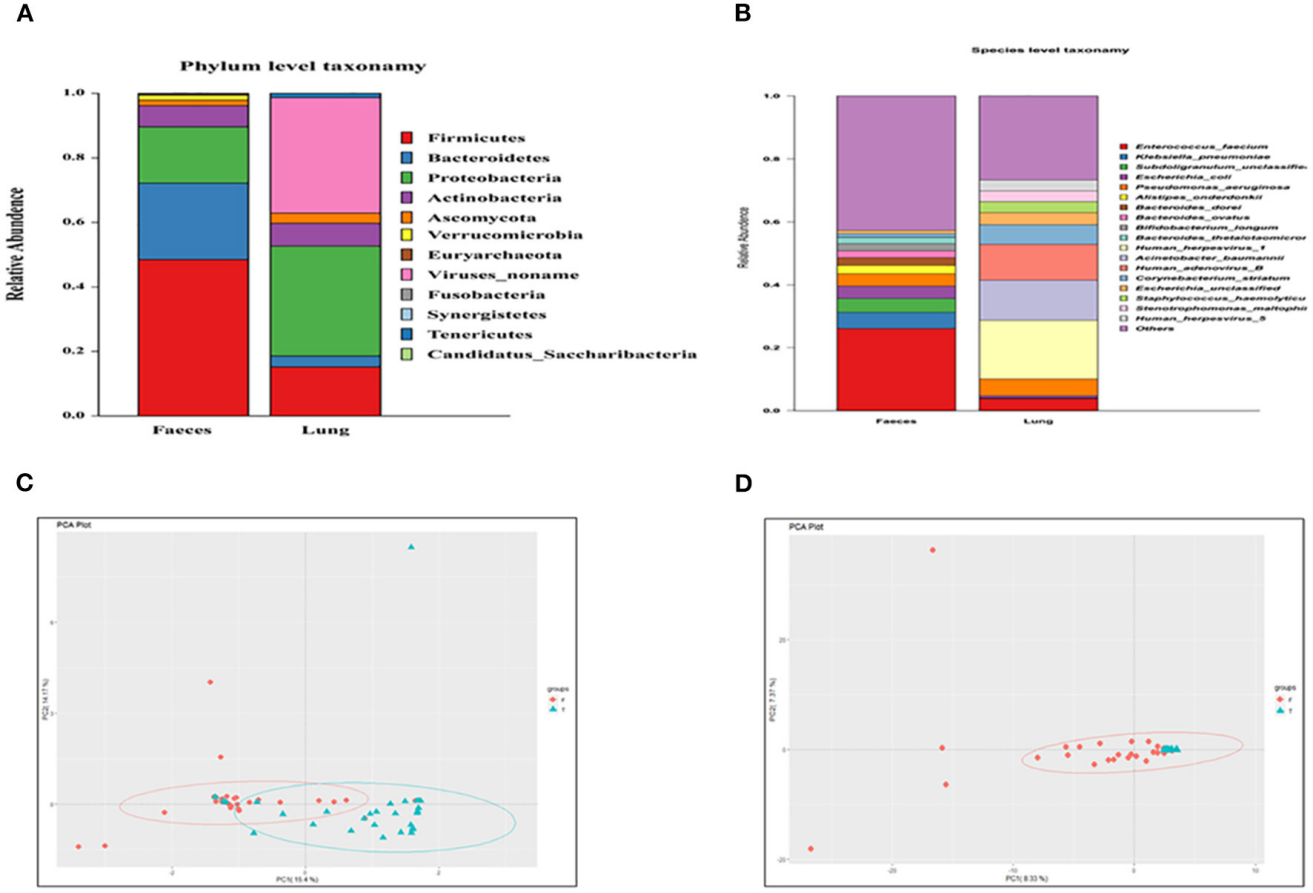
**(A)** Composition of the lung and fecal microbiome at the phylum level; **(B)** composition of the lung and fecal microbiome at the species level; **(C)** Principal component analysis (PCA) of the phylum; and **(D)** PCA of the species.

Metagenomic analysis at the phylum level showed that Firmicutes are the most abundant component in the gut, and the relative abundance of Firmicutes is also high in the lungs of patients with critical illness. Viruses_noname and Proteobacteria were the two most abundant phyla in the lung (Figure 1A).

Metagenomic analysis at the species level showed that *Entrococcus facium* was the main composition in both the gut and lung. In addition, the relative abundance of human herpesvirus was high in the lung (Figure 1B).

In the PCA of phyla (Figure 1C), the distances of samples in the lung were relatively close. In addition, the lung and gut microbiome showed no distinct separation by the PC1, which accounted for 15.4% of the total variation. PC2 accounted for 14.17% of the variance in the bacterial communities. Overall, PC1 and PC2 explained 29.57% of the variation between the different bacterial communities.

In the PCA of species (Figure 1D), the lung and gut microbiomes were also not separated by the PC1 that accounted for 8.33% of the total variation. PC2 accounted for 7.37% of the variance in the bacterial communities. Overall, the PC1 and PC2 axes explained 15.7% of the variation between the different bacterial communities. PCA analyses indicated similar components of the lung and gut microbiome.

### Common Species Analysis Between Lung and Fecal Microbiome

**Figure 2:**
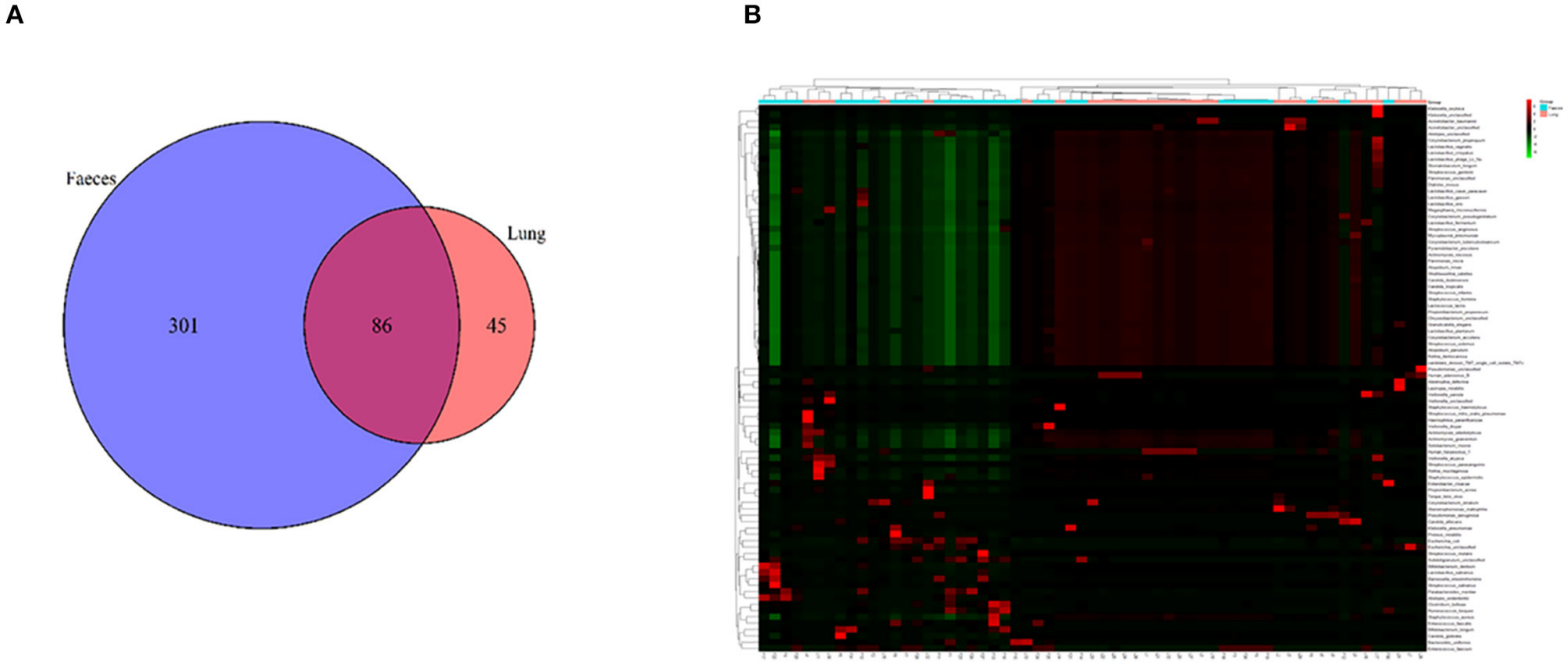
**(A)** A Venn diagram of the microbiome of the lung and gut. **(B)** Cluster analysis of the microbiome in the lung and gut. F, feces; L, lung.

A Venn diagram was created to show the bacteria shared or unique to BALF and feces. A total of 86 species were common between BALF and fecal samples (Figure 2A).

We performed clustering to analyze the composition of the microbiome in both the lung and gut. A cluster analysis revealed that the composition of the microbiome in the lung was partially similar to that in the fecal samples. Some samples in the lung and fecal shared the same composition (Figure 2B).

### Correlation Between Immunity and Microbiome in Patients With Critical Illness

**Figure 3:**
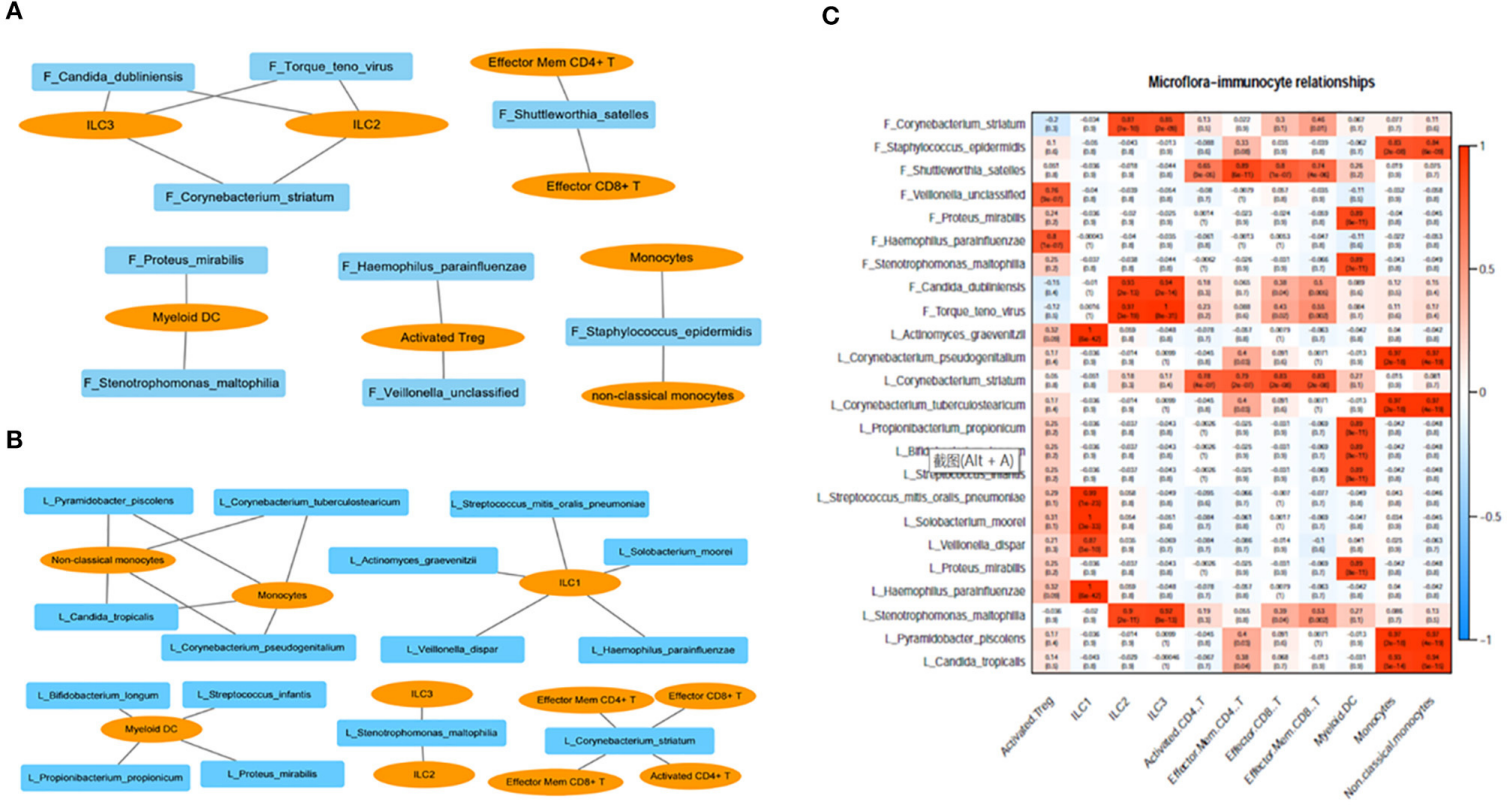
**(A)** Correlation between immunocytes and the microbiome in the gut; **(B)** correlation between immunocytes and the microbiome in the lung; **(C)** correlation between immunocytes and the microbiome in the lung and gut. F, feces; L, lung.

We analyzed the correlation between immunocytes and the microbiome in the lung and gut. *C. striatum* in the lung turned out to be significantly correlated with CD8+T cell and CD4+T cell included effector CD8+ T, activated CD4+ T, effector memory CD4+ T, and effector memory CD8+ T. Meanwhile, *C. striatum* in the gut has a significant correlation with Innate lymphoid cell 2 (ILC2) and Innate lymphoid cell 3 (ILC3). In addition, a correlation analysis showed that *Stenotrophomonas maltophilia* in the lung had a significant correlation with ILC2s and ILC3s, while *S. maltophilia* in the gut showed a significant correlation with myeloid dendritic cells (DCs). Monocytes had a significant correlation with *Staphylococcus epidermidis* in the gut and showed a correlation with *Pyramidobacter piscolens, Corynebacterium tuberculostearicum, Candida tropicalis*, and *Corynebacterium pseudogenitalium* in the lung (Figures 3A–C).

### Strain Level Analysis of *C. striatum*

**Figure 4:**
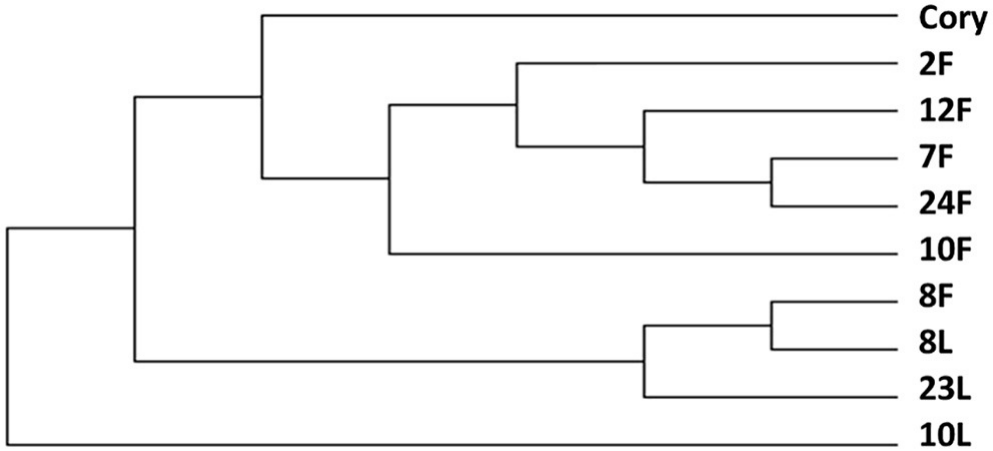
Strain level analysis of *Corynebacterium striatum*. The *C. striatum* strain of samples of the 8# patient was similar. F, feces; L, lung.

The change in strain level is the key factor in determining the function of the microbiome. Our study showed that *C. striatum* was significantly correlated with the immunity of patients with critical illness in the lung and gut. Therefore, we performed a strain-level analysis of *C. striatum*. The results showed that the strain of samples of the 8^#^ patient was similar. An evolutionary tree revealed that *C. striatum* in the lung might trace back to the gut (Figure 4).

## Discussion

The microbiome is considered to be related to human disease and health (15, 16). Illness might cause changes in the microbiome, and microbiome changes might be one of the pathogeneses of many diseases (17, 18). Patients in RICU are bedridden. Diet changes, antibiotic use, and mechanical ventilation could change the balance of the microbiome. The lung-gut axis refers to the physiological and pathological interaction of the lung and gut in the process of diseases (6). The microbiome of lung-gut axis is recently concerned. In our study, we used metagenomics to analyze the lung microbiome and gut microbiome of patients with critical illness in the RICU. We revealed the correlation and difference in microbiome composition between the lung and gut in patients with critical illness at the phylum and species levels. There were 86 common species in both the lung and gut. Human herpesvirus is the two most common species in the lung microbiome, and *Enterococcus faecium* is the most common species in the gut microbiome. In addition, the abundance of *E. faecium* was relatively high in the lung. Our study showed that the abundance of *E. faecium* is high in both the lung and gut of patients with critical illness in the RICU. *E. faecium* is a commensal bacterium of the human gastrointestinal tract (19). Microbiome translocation might be the mechanism that *E. faecium* is high in both the lung and gut in patients with critical illness. Robert P Dickson found out that the lower gastrointestinal tract might be the likely source community of lung bacteria in patients with critical illness (8). The results revealed the partial similarity between the lung and gut in the composition of the microbiome, which provided possible evidence of the lung-gut axis in the microbiome.

We found that lung microbiome was correlated with gut microbiome to a certain extent which might reflect the field of lung-gut axis in the microbiome. As one aspect of lung-gut axis, the microbiome in the lung and gut has been proven to be related to immunity through microbial-related molecules and metabolites produced by microbiota (6). Therefore, immunity with lung microbiome and gut microbiome could partly demonstrate the relation of immunity and lung-gut axis.

The human microbiome is considered a major modulator of the immune system in health and disease (20). Our study indicated that *C. striatum* in both the lung and gut of patients with critical illness were closely related partial immune cells (*C. striatum* in the lung correlated with CD8+T cell and CD4+T cell included effector CD8+ T, activated CD4+ T, effector memory CD4+ T, and effector memory CD8+ T; *C. striatum* in the gut correlated with ILC2 and ILC3). *C. striatum* is a Gram-positive, nonsporulating rod and commensal colonizer of the skin and mucous membranes (21). Metagenomic analysis of the lung and gut microbiome in patients with critical illness showed that the abundance of *C. striatum* was relatively high in both the lung and gut. Interestingly, *C. striatum* in the lung and gut showed a correlation with different immune cells. Previous studies have confirmed that the microbiome usually affects the body's immunity in two ways: one is microbial-related molecules; the other is the related metabolites produced by microbiota (6). Besides, the metabolites of gut microbiota turned out to be related with the immune-mediated pathway which regulated the intestinal Niemann-Pick C1-like 1 expression (22). This result indicated that the same antigen may cause different immune responses in different parts of the human body. In the current clinical work, the detection of immune cells and other immune functions cannot accurately judge whether it is related to a certain bacterium. It can only help to show the general diagnosis direction of the pathogen. The diagnosis of clinical pathogens needs to be combined with other clinical evidence, such as bacterial culture and bacterial smear. In the future, we could expand the sample size and make further efforts to clarify the correlation between certain pathogens and immunity. In this way, we could determine the presence of certain pathogens through the changed number of immune cells and improve the diagnostic accuracy of certain pathogens. The mechanism of this phenomenon needs to be further explored.

The lung-gut axis has been proven to be possible pathogenesis in many diseases (23, 24). Metagenomic analysis of the lung and gut microbiome of patients with critical illness showed that *C. striatum*, one of the common bacteria between the lung and gut, is closely related to immunity. Strain-level analysis revealed that in some patients, *C. striatum* in the lung and gut might share the same source, supporting the concept of a gut-lung axis in humans. There were many mechanisms of correlation between lung and gut, such as microbiome translocation. The Lung-gut axis turned out to be the potential pathogenesis of many diseases (25, 26). The role of lung-gut axis is reflected in many aspects and the microbiome is just one aspect of the lung-gut axis (27). Seeking out the characteristic of microbiome of the lung-gut axis might be helpful in the diagnosis and prognosis in diseases that are correlated with the lung-gut axis. Besides, in most cases, the samples of gut are easier to obtain than that of the lung. If we can understand the correlation between lung and gut in the microbiome, it is possible to infer the composition of lung microbiome from the gut, which might optimize the clinical diagnosis process.

## Limitation

There are some limitations in our study. First, there were only 31 paired lung-gut-blood samples. The sample size was relatively small. However, there are not so many reports on the use of metagenomic sequencing to describe the microbiome of lungs and gut. From the perspective of metagenome sequencing, the sequencing is deeper and more accurate (28). Second, our study only included the patients in RICU and we strictly control the indications and contraindications of tracheoscopy and collect BALF after obtaining informed consent. Whereas, we did not include the healthy control. Based on the reality of clinical work, BALF collection is an invasive procedure with certain risks. It is difficult to collect BALF from a healthy control. Although we have not enough samples in this experiment, the correlation analysis of immunity with the microbiome in the lung-gut axis may indicate the potential diagnostic and therapeutic targets of patients with critical illness. We will improve the research plan in the future and seriously consider the rigorous inclusion of healthy control. Besides, the possibility of impact due to hospital-derived pathogens cannot be absolutely avoided in the current study. Therefore, in future research, we should further formulate strict standards for the process of collecting samples. The samples of operating instruments, containers, patient ward environment, operator skin, doctors, nurses, and other people or objects should be collected so that we can check and determine whether the samples were influenced by the source of hospital-acquired pathogens.

## Conclusions

We used metagenomics to analyze the lung and gut microbiome of patients with critical illness in the RICU. The results revealed that the microbiome in the lung and gut showed a correlation to some extent, and the *C. striatum* of some patients in the lung and gut shared the same source, supporting the concept of a gut-lung axis. In addition, the microbiome showed a correlation with immunity, indicating a potential therapeutic target in patients with critical illness.

## Data Availability Statement

The datasets presented in this study can be found in online repositories. The name of the repository and accession number can be found below: National Genomic Data Center; PRJCA007879 (https://ngdc.cncb.ac.cn/bioproject/browse/PRJCA007879).

## Ethics Statement

The studies involving human participants were reviewed and approved by Ethic Committee of Chinese PLA General Hospital. The patients/participants provided their written informed consent to participate in this study.

## Author Contributions

BL and YY are the guarantors of the paper and realized statistics. LX and JW designed the study. MZ, NZ, and JC analyzed the data. KX and PY ensured the inclusion of patients. ZD, PY, KW, and KX collect clinical samples. All authors contributed to the article and approved the submitted version.

## Funding

This work was supported by the Key Projects of Military Logistics Scientific Research Program (BLB18J008) and China NSFC Grant (82172109).

## Conflict of Interest

MZ was employed by the company GE Healthcare. The remaining authors declare that the research was conducted in the absence of any commercial or financial relationships that could be construed as a potential conflict of interest.

## Publisher's Note

All claims expressed in this article are solely those of the authors and do not necessarily represent those of their affiliated organizations, or those of the publisher, the editors and the reviewers. Any product that may be evaluated in this article, or claim that may be made by its manufacturer, is not guaranteed or endorsed by the publisher.
